# Different Lipid Regulation in Ovarian Cancer: Inhibition of the Immune System

**DOI:** 10.3390/ijms19010273

**Published:** 2018-01-17

**Authors:** Christina Wefers, Tjitske Duiveman-de Boer, Petra L. M. Zusterzeel, Leon F. A. G. Massuger, David Fuchs, Ruurd Torensma, Craig E. Wheelock, I. Jolanda M. de Vries

**Affiliations:** 1 Department of Tumor Immunology, Radboud Institute for Molecular Life Sciences, Radboudumc, Geert Grooteplein Zuid 28, 6525 GA Nijmegen, The Netherlands; christina.wefers@radboudumc.nl (C.W.); tjitske.duiveman-deboer@radboudumc.nl (T.D.B.); ruurd.torensma@radboudumc.nl (R.T.); 2 Department of Obstetrics and Gynecology, Radboudumc, Geert Grooteplein 10, 6525 GA Nijmegen, The Netherlands; petra.zusterzeel@radboudumc.nl (P.L.M.Z.); leon.massuger@radboudumc.nl (L.F.A.G.M.); 3 Division of Physiological Chemistry II, Department of Medical Biochemistry and Biophysics, Karolinska Institutet, Scheeles Väg 2, SE-171 77 Stockholm, Sweden; david.fuchs@ki.se (D.F.); craig.wheelock@ki.se (C.E.W.)

**Keywords:** lipid intermediates, paralyzed T cells, modified lipid metabolism, tumor microenvironment

## Abstract

Lipid metabolism is altered in several cancer settings leading to different ratios of intermediates. Ovarian cancer is the most lethal gynecological malignancy. Cancer cells disperse in the abdominal space and ascites occurs. T cells obtained from ascites are unable to proliferate after an antigenic stimulus. The proliferation of ascites-derived T cells can be restored after culturing the cells for ten days in normal culture medium. No pathway aberrancies were detected. The acellular fraction of ascites can inhibit the proliferation of autologous as well as allogeneic peripheral blood lymphocytes, indicating the presence of soluble factors that interfere with T cell functionality. Therefore, we analyzed 109 lipid mediators and found differentially regulated lipids in suppressive ascitic fluid compared to normal abdominal fluid. Our study indicates the presence of lipid intermediates in ascites of ovarian cancer patients, which coincidences with T cell dysfunctionality. Since the immune system in the abdominal cavity is compromised, this may explain the high seeding efficiency of disseminated tumor cells. Further research is needed to fully understand the correlation between the various lipids and T cell proliferation, which could lead to new treatment options.

## 1. Introduction

Worldwide, more than 239,000 women per year are diagnosed with ovarian cancer and 152,000 succumb annually [1,2]. Due to aspecific symptoms, the majority of the patients present in an advanced stage, having extensive tumor burden and often accumulation of fluid, called ascites, in the peritoneal cavity. Ascitic fluid contains lymphocytes, mesothelial cells, and macrophages; tumor cells and; in the case of rapid fluid development, a high proportion of red blood cells. The ratio of the different cells can vary between patients, but lymphocytes are generally the biggest cell population [3]. T cell immunity in the tumor microenvironment has a profound impact on ovarian cancer outcome and plays a significant role in controlling recurrence [4,5]. Several studies demonstrated that high levels of CD8^+^ T cells positively correlate with patient survival in high-grade serous ovarian cancer [6,7,8]. However, tumor cells can escape destruction by the immune system in several ways. Ovarian cancer cells can recruit and induce the expansion of regulatory T cells (T_regs_), which are able to inhibit the anti-tumor response of effector CD8^+^ T cells, either by secretion of interleukin-10 (IL-10) and transforming growth factor-β (TGF-β) or via a cell-cell contact-dependent mechanism. Accumulation of T_regs_ at the tumor site is associated with reduced survival of ovarian cancer patients, as are high CD4/CD8 ratios. Furthermore, CD8^+^ effector T cells are inhibited by tumor macrophages expressing B7-H4 [9,10]. Recent studies also investigated the effect of the presence of immune cells in ascites and confirmed the results from solid ovarian cancer [11]. Patients with a high CD4/CD8 ratio have a poorer outcome, whereas a high tumor infiltrating lymphocytes (TIL)/T_reg_ ratio is generally favorable [10].

Over the past 30 years, the standard treatment for ovarian cancer has remained cytoreductive surgery in combination with platinum- and taxane-based chemotherapy (carboplatin and paclitaxel) [12,13]. About 70% of the patients respond to the first-line therapy, although most patients will get recurrent disease within two years [14,15]. Therefore, new treatment options are necessary. Enforcing the immune response through immunotherapy could benefit ovarian cancer patients. However, ascites provides a growth-promoting environment for tumor cells by down-regulation of the anti-tumor immune response. Understanding the immunosuppressive mechanisms in ovarian cancer is essential for effective enhancement of the immune system. To determine the functionality of immune cells in the peritoneal cavity of ovarian cancer patients, we measured the proliferation and cytokine secretion of ascites-derived T cells. In this paper, we demonstrate that ascites can transiently suppress local T cell proliferation in the peritoneal cavity, weakening the role of the immune system. This suppression seems to be mediated by a deregulated lipid metabolism in malignant ascites. Lipid mediators are known to modulate the immune response in cancer [16,17,18]. Low oxygen in the tumor microenvironment limits the aerobic glycolysis and thus proliferation of T cells but this is overcome by switching to fatty acid oxidation [19]. In several cancers, lipooxygenases (LOX) are modified which lead to either pro-tumorigenic or anti-tumorigenic functions [20]. Interfering with lipid metabolism might open new ways to help the immune system to eradicate tumor cells.

## 2. Results

### 2.1. Ascites-Derived T Cells Proliferate Poorly upon αCD3/28 Stimulation

**Figure 1:**
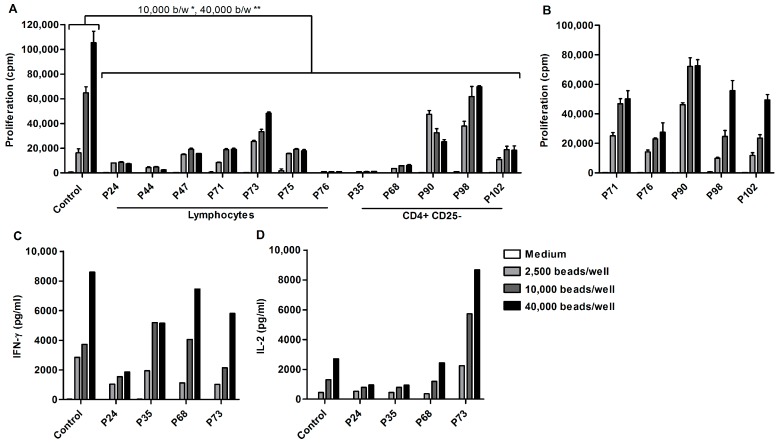
Decreased proliferation, but normal cytokine secretion of ascites-derived T cells. Proliferation and cytokine secretion was measured after stimulating 5 × 10^4^ cells with increasing concentrations of αCD3/28 beads for three days. (**A**) Proliferation of control PBLs (mean of *n* = 8), sorted lymphocytes (*n* = 7) and sorted CD4^+^CD25^−^ T cells (*n* = 5) from ascites-derived MNCs. (**B**) Proliferation of patient-derived PBMCs. Data are given as mean of cpm ^3^H-thymidine incorporation of triplicate cultures ± SEM. (**C**) IFN-γ; and (**D**) IL-2 production of ascites-derived lymphocytes (*n* = 4) and control PBLs (*n* = 3). Supernatants of triplicate cultures were pooled and tested by an 11-plex for cytokine production. Statistical analysis using one-way ANOVA with Bonferroni post-test (* *p* < 0.05, ** *p* < 0.01).

A functional cellular immune response is crucial for the eradication of tumor cells. One of the key processes of cellular immunity is antigen specific T cell proliferation. Therefore, lymphocytes from cryopreserved ascites-derived mononuclear cells (MNCs) were sorted using a lymphocyte gate and stimulated with different concentrations of αCD3/28 beads. The proliferative capacity was measured after three days (Figure 1A).

Peripheral blood lymphocytes (PBLs) from healthy individuals had higher proliferation rates when stimulated with increasing concentrations of αCD3/28 beads. In contrast, the majority of ascites-derived T cells hardly proliferated upon αCD3/28 stimulation. Only two patients (P73 and P98) showed a dose–response curve when stimulated with increasing concentration of beads, but proliferation was low compared to PBLs from healthy individuals. Altogether, proliferation of ascites-derived cells was significantly lower when stimulated with 10,000 or 40,000 beads/well compared to PBLs from control individuals (mean difference 50,737 cpm and 63,549 cpm, *p* < 0.05 and *p* < 0.01 respectively). The decreased proliferation of ascites-derived T cells was not mediated by the presence of T_regs_, as there was no significant difference in the proliferation of sorted CD4^+^CD25^−^ T cells lacking T_regs_ and sorted lymphocytes from ascites that contain T_regs_.

To rule out a systemic T cell defect, the proliferative capacity of patient peripheral blood mononuclear cells (PBMCs) was tested (Figure 1B). Patient PBMCs showed enhanced proliferation when stimulated with increasing concentrations of αCD3/28 beads. Furthermore, the proliferation of patient PBLs did not differ significantly from control PBLs (mean difference 31,726 cpm, *p* ≥ 0.05).

Lack of cytokine production could be the cause of T cell proliferation insufficiency. Therefore, the cytokine secretion was measured after three days of αCD3/28 bead stimulation. Using an 11-plex Flowcytomix Multiplex assay, samples from four patients and three controls were analyzed for the secretion of interferon γ (IFN-γ), interleukin-2 (IL-2), IL-4, IL-5, IL-6, IL-10, tumor necrosis factor α (TNF-α), and tumor necrosis factor β (TNF-β).

Ascites-derived T cells from all tested patients showed an increase in IFN-γ and IL-2 production when stimulated with increasing concentrations of αCD3/28 beads (Figure 1C,D). Moreover, there was no significant difference in the IFN-γ and IL-2 production of ascites-derived cells and control PBLs. All other cytokines were produced at low levels.

### 2.2. Ascites-Derived CD4^+^CD25^-^ T Cells Are Unresponsive to IL-2

**Figure 2:**
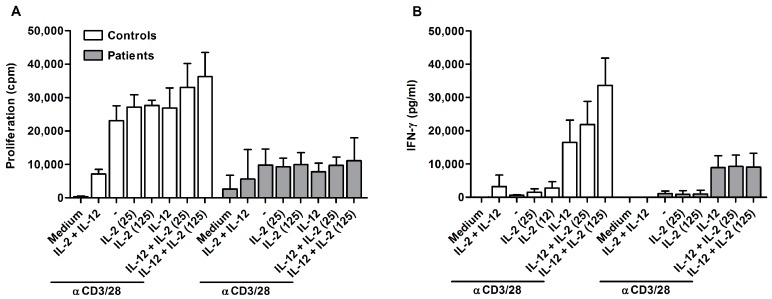
Hypoproliferation of ascites-derived T cells cannot be overcome by IL-2 or IL-12. (**A**) Proliferation; and (**B**) IFN-γ secretion of control (*n* = 3) and ascites-derived CD4^+^CD25^−^ T cells (P61, P68, P73) after three days of stimulation. Cells were cultured with αCD3/28 (2500 beads/well), IL-2 (25 or 125 U/mL), and IL-12 (1000 pg/mL), or a combination of the different stimuli. Values are means ± SD.

One possible explanation for the hampered proliferation of ascites-derived lymphocytes is T cell anergy. T cells can become functionally inactivated or anergic after antigen encounter, meaning that they do not react to re-stimulation. To test this hypothesis, CD4^+^CD25^-^ ascites-derived T cells were stimulated for three days with αCD3/28 beads in combination with IL-2 and/or IL-12. IL-2 is known for its ability to overcome the non-proliferative status of anergic T cells. IL-2 and IL-12 synergize [21] and are able to reverse T cell anergy in mycobacterial disease [22]. Subsequently, proliferation as well as IFN-γ secretion was measured (Figure 2). 

Stimulation with αCD3/28 resulted in proliferation of control cells (Figure 2A). The proliferation was enhanced when cells were treated with αCD3/28 in combination with IL-2 and/or IL-12. In contrast, ascites-derived cells barely proliferated in response to αCD3/28 beads. Stimulation with αCD3/28 in combination with IL-2 and/or IL-12 was not able to overcome the unresponsiveness of patient-derived cells. Similar effects were observed for IFN-γ secretion (Figure 2B). Control cells produced low amounts of IFN-γ when stimulated with αCD3/28 beads only. Addition of IL-2 resulted in an increase of IFN-γ secretion, which was even stronger for stimulation with αCD3/28 beads together with IL-12. There was a synergistic effect on IFN-γ secretion when cells were stimulated in the presence of IL-2 and IL-12. This synergistic effect was absent in patients samples. Ascites-derived cells did not increase IFN-γ when stimulated in the presence of IL-2. Cytokine secretion was only enhanced in response to IL-12. This indicates that ascites-derived T cells are unresponsive to IL-2.

### 2.3. Ascites-Derived CD4^+^CD25^-^ T Cells Have a Normal Expression of IL-2R and Its Downstream Signaling Components

**Figure 3:**
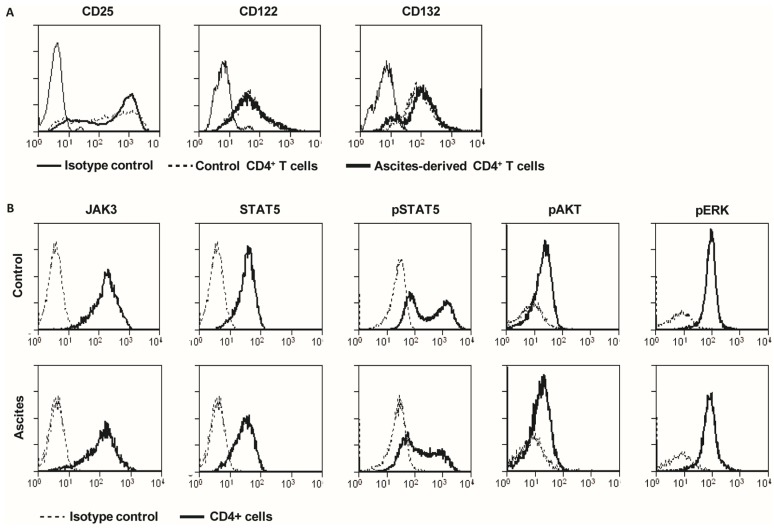
Normal expression and phosphorylation of the IL-2 receptor and its downstream signaling components. PBLs of healthy individuals or sorted ascites-derived CD4^+^CD25^−^ T cells (5 × 10^5^ cells/well) were stimulated with 20,000 αCD3/28 beads/well for three days. Subsequently, samples were stained for flow cytometry analysis. (**A**) Cells were stained for isotype control, CD25, CD122, and CD132. Plots are representative for eight controls and three patients. (**B**) Control (*n* = 3) and ascites-derived T cells (*n* = 3) were stained for JAK3, STAT5, phospho-STAT5, phosphor-AKT and phosphor-ERK. One representative example is shown.

We hypothesized that the unresponsiveness to IL-2 could be caused by aberrant expression of the high affinity IL-2 receptor (IL-2R). Therefore, expression of the three IL-2R subunits CD25, CD122, and CD132 was measured on stimulated ascites-derived CD4^+^CD25^−^ T cells [23]. All three components were expressed on both, control PBLs and on ascites-derived T cells (Figure 3A). In each patient, there was a fraction of cells that highly expressed CD25, and this level was comparable to control PBLs.

Since the high affinity IL-2R was normally expressed, we reasoned that the cause of IL-2 unresponsiveness could be located downstream in the IL-2 signaling pathway. IL-2 binding to its receptor results in Janus kinase 3 (JAK3) phosphorylation. Next, three different signaling pathways can be activated, via signaling of signal transducer and activator of transcription 5 (STAT5), phosphoinositide 3-kinase (PI3K) or through mitogen-activated protein kinase (MAPK). Therefore, total JAK3 and STAT5 was measured, as well as phosphorylation of STAT5, the serine/threonine kinase AKT and the MAP kinase ERK1/2 (Figure 3B). Previous studies showed a reduced expression of JAK3 as a mechanism for IL-2 non-responsiveness [24]. Ascites-derived lymphocytes from ovarian cancer patients normally expressed JAK3 and STAT5. Next, the STAT5 phosphorylation was evaluated by intracellular flow cytometry. Freshly isolated ascites-derived MNCs and PBMCs from the same patient were stained for CD4, incubated with or without 100 U/mL IL-2 for one hour, and subsequently stained for phospho-STAT5. IL-2 stimulated samples had a higher percentage of phospho-STAT5 positive CD4 cells compared to medium-treated controls. The size of CD4^+^ and phospho-STAT5 positive population differed greatly among patients, ranging from 19–63% in the ascites-derived cells to 26–50% in the corresponding PBLs. To analyze the phosphorylation of ERK1/2 and AKT, ascites-derived lymphocytes were stimulated with αCD3/28 for 4 days. Control PBLs and ascites-derived lymphocytes were highly positive for CD25 and had comparable levels of phosphorylated AKT and ERK1/2.

### 2.4. Acellular Fraction of Ascites Inhibits Proliferation of Control PBLs

**Figure 4:**
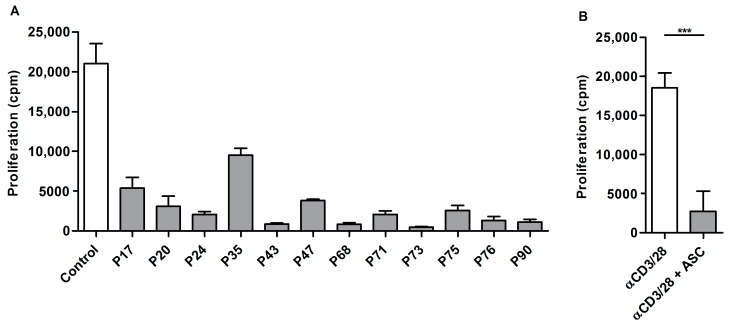
Ascites inhibits proliferation of PBLs from healthy donors: (**A**) 5 × 10^4^ control PBLs were stimulated with 2500 beads/well in the presence of 20% ascites fluid from ovarian cancer patients. Data are given as mean of cpm ^3^H-thymidine incorporation of triplicate cultures ± SEM. (**B**) Summary of PBLs stimulated with αCD3/28 (*n* = 4) and PBLs stimulated in the presence of ascites (*n* = 14). Values are given as mean ± SD. Statistical analysis using unpaired student’s *t*-test with Welch’s correction (*** *p* < 0.001).

The tumor microenvironment can strongly influence T cell activation. As we observed a severely decreased proliferative capacity in ascites-derived T cells, we wondered if ascites could induce the same effect in control PBLs. Lymphocytes obtained from healthy donors were stimulated with αCD3/28 beads in the presence of the acellular fraction of ascites and proliferation was measured after three days. Some variability in the inhibition of proliferation was observed. However, the proliferative capacity of PBLs was markedly decreased in the vast majority of tested donors (Figure 4).

### 2.5. Suppression of Ascites-Derived T Cells Is Reversible

**Figure 5:**
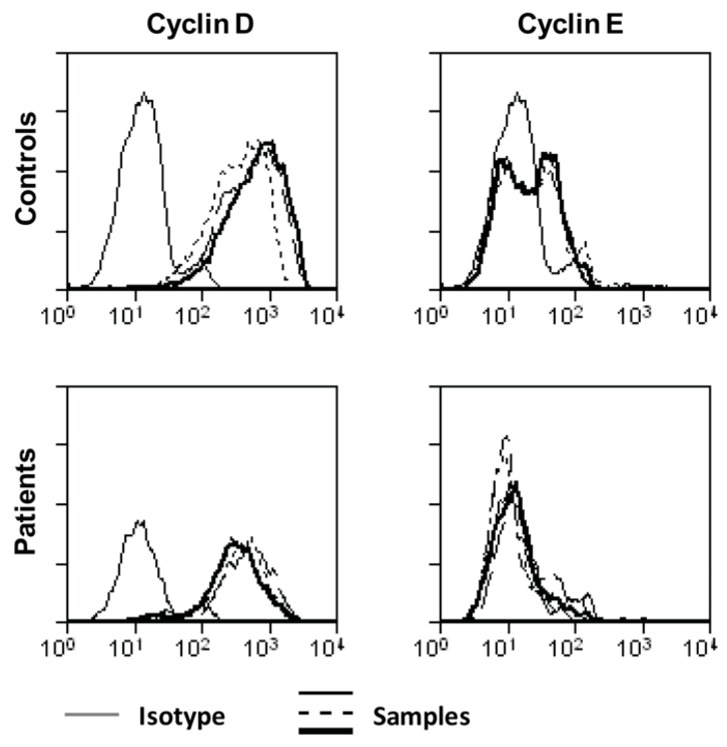
Cyclin D and E expression in control and ascites-derived lymphocytes. Control PBLs or sorted ascites-derived lymphocytes (5 × 10^5^ cells) were stimulated with 20,000 αCD3/28 beads/well. After 48 h, cells were stained for CD4, cyclin D and cyclin E. Plots are representative for three individual controls and four patients.

As IL-2 signaling was not (or only minimally) affected in ascites-derived T cells, we hypothesized that there could be a more general proliferation defect. We therefore decided to analyze the expression of cyclin D and cyclin E, two proteins required for the transition from G_1_- to S-phase. Lymphocytes isolated from ascites of ovarian cancer patients were unable to upregulate cyclin E upon stimulation (Figure 5).

To determine whether the proliferative capacity of ascites-derived T cells can be restored, sorted lymphocytes were stimulated with αCD3/28 beads for three days. Subsequently, a fraction of those cells was cultured for 10 days in the presence of IL-2 and IL-7. After 10 days, the cells were washed and re-stimulated for three days with αCD3/28 in the presence or absence of IL-2, IL-12, or a combination of both cytokines.

**Figure 6:**
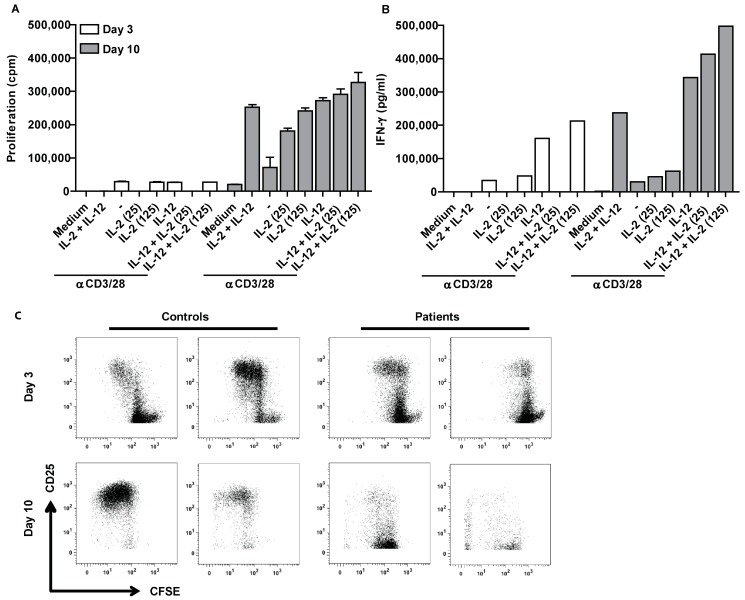
IL-2 and IL-12 responsiveness of ascites-derived CD4^+^CD25^-^ can be restored. In total, 5 × 10^5^ sorted CD4^+^CD25^-^ ascites-derived T cells of P71 were stimulated directly with 5000 αCD3/28 beads/well for three days in the presence or absence of 25 or 125 U/mL IL-2, 1000 pg/mL IL-12 or a combination of both and subsequently cultured for 10 days in normal medium before being re-stimulated. (**A**) Proliferation was measured before (Day 3) and after culture (Day 10). Data are given as mean of cpm ^3^HTdR incorporation of triplicate cultures ± SEM. (**B**) Triplicate cultures were pooled and tested in three different dilutions in an IFN-γ ELISA. The data are representative for three tested patients. (**C**) Ascites-derived MNCs were sorted using a lymphocyte gate. Subsequently, ascites-derived lymphocytes and PBLs from healthy donors were labeled with CFSE, plated at a concentration of 5 × 10^5^ cells and stimulated with 20,000 αCD3/28 beads. After three days, the cells were stained for CD4 and CD25 and measured with FACS (upper panel). A part of the stimulated cells was kept in culture for ten days and restimulated with αCD3/28 beads, stained for CD4 and CD25 and subsequently measured with FACS (lower panel).

Ascites-derived lymphocytes barely proliferated in response to the first stimulation with αCD3/28 beads, alone or in combination with IL-2 and IL-12 (Figure 6A). Additionally, IFN-γ secretion was also low across all treatment conditions (Figure 6B). However, when cells of the same patient were re-stimulated after 10 days of culturing in the absence of ascites, proliferation and cytokine secretion was markedly increased and comparable to control PBLs from healthy donors. Together, this indicates that the proliferation halt of ascites-derived T cells is reversible.

Ascites-derived lymphocytes and PBLs from healthy individuals were furthermore labeled with CFSE and subsequently stimulated with αCD3/28 beads. After three days, a small fraction of the samples was stained for CD4 and CD25 and the proliferative capacity was analyzed by flow cytometry. The remaining cells were kept in culture without ascites. After 10 days, the CFSE positive cells that did not divide were sorted and re-stimulated with αCD3/28 beads for three days, stained for CD4 and CD25 and analyzed by flow cytometry to determine if the proliferative capacity of those cells was restored. Indeed, the initially non-proliferative cells showed proliferation after re-stimulation with αCD3/28 (Figure 6C).

### 2.6. Altered Lipid Metabolites in Suppressive Ascites

Our results point toward a suppressive compound within ascites. Lipid metabolism is deregulated in many cancers and known to affect the immune system in various ways. Therefore, normal abdominal fluid and suppressive ascites fluid were screened for 109 lipid mediators using LC-MS/MS (Table 1).

Ascites from five patients with maximal suppressive ascites and abdominal fluid from five control individuals was analyzed. We expected only a few lipid mediators to be different. Instead, 82 metabolites were differentially expressed in ascites compared to healthy abdominal fluid controls. The results were consistent across controls and patients. The values for each lipid metabolite were averaged and the ratio of the concentration in ascites and normal abdominal fluid was calculated.

**Figure 7:**
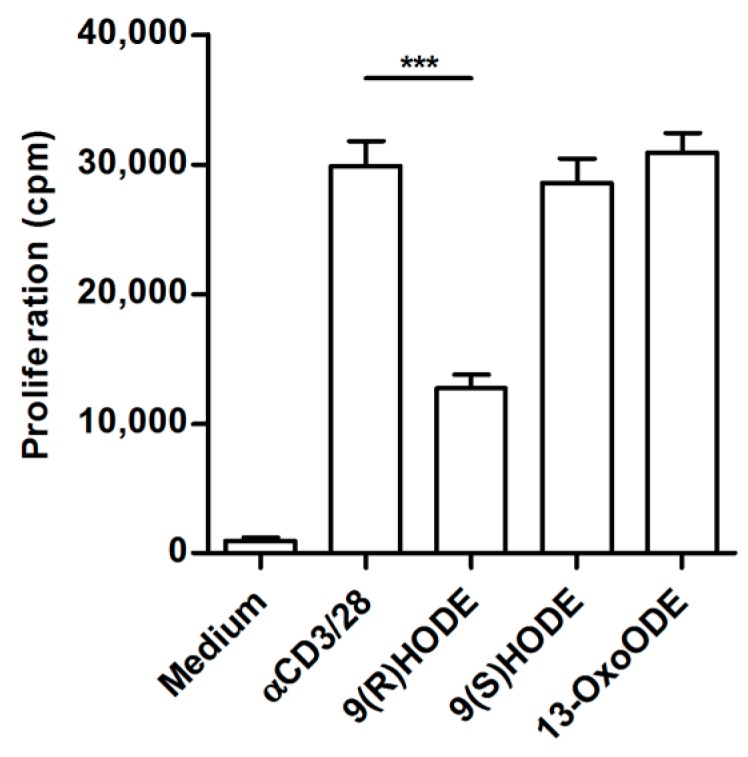
Synthetic Lipid-metabolites partially inhibit T cell proliferation. In total, 50,000 control PBLs from healthy donors were stimulated with αCD3/28 for three days in the presence of 9(R)HODE, 9(S)HODE or 13-OxODE. Only 9(R)HODE decreased the proliferation of control PBLs. Data given as mean of cpm ^3^HTdR incorporation of triplicate cultures ± SEM. Statistical analysis was performed using one-way ANOVA with Bonferroni post-test (*** *p* < 0.001).

Several of the lipid metabolites are able to activate peroxisome proliferator-activated receptors (PPAR), which can modulate the activity of immune cells [25]. PPAR can be activated by 9-HODE, a lipid metabolite that was increased in suppressive ascites. We tested purified 9-HODE isomers in a T cell activation assay and indeed 9(R)HODE was able to partially inhibit proliferation, while 9(S)HODE and 13-OxODE did not have an effect on proliferation (Figure 7).

## 3. Discussion

Epithelial ovarian cancer is a devastating disease. Most patients are diagnosed at an advanced stage and present with a massive tumor burden. The immune system plays an important role in cancer development. Ovarian cancer metastases are generally found within the peritoneal cavity and barely elsewhere in the body [26,27]. CD8^+^ T cell infiltrates in primary ovarian cancer and ovarian cancer-associated ascites are correlated to improved prognosis and survival [8]. Despite their presence, complete tumor regression is not observed, pointing towards suppression of a full blown immune response [19,28]. Our findings demonstrate that ascites fluid is able to locally and transiently inhibit T cell proliferation within the peritoneal cavity, creating a deficiency in T-cells. In contrast, cytokine secretion of ascites-derived T cells was not affected. The fact that ascites fluid decreases the proliferative capacity of control PBLs points towards a soluble factor present in ascites that interferes with T cell proliferation. Addition of exogenous IL-2 and IL-12 did not increase proliferation of ascites-derived T cells. Since patient-derived cells produced more IFN-γ when stimulated with IL-12, we reasoned that IL-2 unresponsiveness might be the cause for the observed hypo proliferation. Several studies described defects in different components of the IL-2R signaling pathway, resulting in decreased T cell proliferation, such as the absence of CD25 expression [29], impaired STAT1 and STAT5 activation [30] or soluble products released from renal cell carcinoma that suppress JAK3 [24]. Interestingly, we could not detect any differences in the expression of IL-2 receptor subunits or the activation of downstream components of the IL-2R signaling pathway in ascites-derived T cells. The next members of the pathway, JAK3 and STAT5, were expressed at normal levels. Furthermore, phosphorylation of STAT5, AKT, and ERK1/2 was detected after stimulation with IL-2 or other stimuli like αCD3/28 beads. We did observe lower levels of cyclin E in ascites-derived lymphocytes, a protein important for transition from G_1_ to S-phase [31]. This suggests that lymphocytes isolated from ascites of ovarian cancer patients were halted in G_0_/G_1_.

Since ascites also inhibited proliferation of normal T cells we analyzed the lipid composition of ascites and normal abdominal fluid. Cancer cells are known to have a deregulated lipid metabolism [32], which could modulate T cell response. Comparison of normal abdominal fluid and ascites from ovarian cancer patients showed that the concentration of several lipid metabolites varies between the two groups. Moreover, these differences were consistent for five high-grade serous ovarian cancer patients and five controls. Since T cells that were isolated from normal abdominal fluid of healthy individuals proliferated normally when kept in standard culture medium, T cell anergy must be either induced by compounds that are only present in suppressive ascites or by metabolites that are highly elevated in suppressive ascites or by lacking intermediates that are present in normal belly fluid. 

Lipid metabolites are bound by peroxisome proliferator-activated receptors (PPARs). The PPARs are the spider in the web that regulates various cell functions depending on the environment and stimulus. Their function is determined by a co-repressor and co-activator bound to PPAR [33]. In T cells, PPAR can bind to nuclear factor of activated T cells (NFAT), thereby inhibiting cell proliferation and IL-2 secretion [25,34]. In our studies, we did not observe lower IL-2 quantities, indicating that NFAT does not bind to activated PPAR. However, another pathway could be interaction of PPAR with other factors like STAT3 or receptor-induced NF-κB that induce p21^WAF/Cip^. P21^WAF/Cip^ in turn modulates p27, resulting in suppression of cyclin E [35]. This could explain the reduced levels of cyclin E that we observed in ascites-derived T cells and thus the lack of proliferation [36]. The lipid metabolite with the highest concentration in suppressive ascites was 5-HETE. However, 5-HETE itself is not able to activate PPAR [37]. It must first be converted to OXO derivatives [25]. Since 5-HETE does not activate PPAR we tested if 9-HODE, another compound that is present in high concentrations in suppressive ascites is able to inhibit cell proliferation. Culturing cells with 9(R)HODE was able to decrease the proliferation of control PBLs. The monocytic cell line U937 is halted in G1 when stimulated in the presence of 9-HODE, most likely via activation of PPARγ [38]. Furthermore, it has been shown that 9-HODE activates G2A, a G protein-coupled receptor, in pro-B and T cells, resulting in accumulation of cells in G2 [39,40]. This indicates that 9-HODE, which is present in large amounts in suppressive ascites, might be able to interfere with T cell proliferation. However, since the inhibition was only partial, several lipids may synergize to block proliferation of ascites-derived T cells. Another candidate could be PGD2, which is low in controls and more than 24 times higher in patient ascitic fluid. PGD2 is converted to 15-deoxy-delta-12,14-PGJ2, a covalent activator of PPAR [41]. 15-deoxy-delta-12,14-PGJ2 is not found in suppressive ascites while it is present in minute amounts in normal belly fluid. Thus far, we cannot decide, which metabolites act in concert with 9-HODE to inhibit T cell proliferation. PPARs are involved in a myriad of other pathways to adapt to changes in the microenvironment. Hopefully, “selective PPAR modulators” (SPPARγ M) can be used to unravel the metabolite(s) [42].

To summarize, we demonstrate that ascites-derived CD4^+^CD25^−^ T cells are impaired in proliferation upon αCD3/28 stimulation. In contrast, cytokine production is not affected. Our data suggest that this could be due to lipid metabolites present in suppressive ascites that cause a proliferation halt of ascites-derived lymphocytes in G_0_/G_1_. Unraveling the mechanisms responsible for the hypo proliferation of ascites-derived T cells remains crucial and should be subject of further investigation, as this may suggest possible treatment options.

## 4. Materials and Methods

### 4.1. Human Subjects

Ascites from stage III or IV epithelial ovarian cancer patients was obtained either during debulking surgery or via ascites drainage. Only material of patients that did not receive chemotherapy prior to sample collection was used in this study. The majority of patients were diagnosed with high-grade serous ovarian cancer, expect for patient 61 and 73 who had mucinous and clear cell ovarian cancer respectively. The guidelines for collection of human material of the Radboudumc were followed. Approval from the Medical Ethics Review Committee was not required. Ascites is considered (RvB13.52245, 28 August 2017) as waste material and the Medical Research Involving Human Subjects Act (WMO) does not apply. Patients were not asked for informed consent and no identifying patient information was used. Ovarian cancer patients donated blood after informed consent, which was taken shortly after surgery. Blood cells from controls were obtained from buffy coats from anonymous healthy donors of the blood bank after informed consent. Experiments were approved and carried out in accordance with the guidelines and regulations of the Radboudumc.

### 4.2. Antibodies

The following antibodies were used in this study: CD4-APC (300514, Beckman Coulter, Brea, CA, USA), CD4-FITC (A07750, Beckman Coulter), CD25-PE (555432, BD Biosciences, San Jose, CA, USA), CD122-PE (IM1978, Beckman Coulter), CD132-APC (338608, BioLegend, San Diego, CA, USA), cyclin D (2936P, Cell Signaling, Danvers, MA, USA), cyclin E (551159, Cell Signaling, Danvers, MA, USA), JAK3 (ab45141, Abcam, Cambridge, UK), STAT5 (9363P, Cell Signaling, Danvers, MA, USA), pSTAT5 (9314S, Cell Signaling, Danvers, MA, USA), pAKT (4075S, Cell Signaling, Danvers, MA, USA), and pERK1/2 (4284S, Cell Signaling).

### 4.3. Cell Isolation and Activation

Ascites was filtered over a 100 µm Cell Strainer (BD Biosciences, San Jose, CA, USA) and centrifuged for 15 min at 1400 rpm. Thereafter, ascites cells were centrifuged over a density gradient. MNCs at the interphase were collected, washed with PBS supplemented with 1% bovine serum albumin, frozen, and stored in liquid nitrogen. Ascites MNCs were thawed, stained for CD4 and CD25, and CD4^+^CD25^−^ T cells sorted with an Epics Altra Cell Sorter (Beckman Coulter, Brea, CA, USA). The purity was always above 95%. In addition, lymphocytes were sorted by setting a lymphocyte gate on the FSC-SSC scatter plot. As a control, either PBMCs from the same patient or PBLs from healthy controls were taken along. PBMCs were obtained by density-gradient centrifugation. To obtain PBLs, PBMCs were plated and monocytes were allowed to adhere to the plastic. After one hour of incubation at 37 °C, the non-adherent PBLs were collected. Cells were plated in a 96 well U bottom plate at a concentration of 5 × 10^4^ cells/well in medium (IMDM, Invitrogen, Thermo Fisher Scientific, Waltham, MA, USA) supplemented with 10% human serum (HS), and stimulated with human T-activator CD3/CD28 Dynabeads^®^ (11131D, Gibco, Thermo Fisher Scientific, Waltham, MA, USA) in the presence or absence of 25, 100 or 125 U/mL IL-2 (Proleukin^®^, Novartis, Arnhem, The Netherlands), 1000 pg/mL IL-12 (219-IL-005, R&D systems, Minneapolis, MN, USA), 25 µg/mL 9(R)-HODE (38405.S4, Bio Connect, Huissen, The Netherlands), 25 µg/mL 9(S)-HODE (38410.S1, Bio Connect, Huissen, The Netherlands) or 25 µg/mL 13-OxODE (38620.S3, Bio Connect, Huissen, The Netherlands) when indicated. Supernatant was collected after three days of incubation prior to the addition of 1 µCi/well of ^3^H-thymidine. After an additional 16 hours of incubation at 37 °C, ^3^H-thymidine incorporation in DNA was measured using a β-counter and expressed as counts per minute (cpm).

### 4.4. Cytokine Measurement

IFN-γ production was assayed using an IFN-γ ELISA. As coating antibody, αIFN-γ clone 2G1 (M700a, Thermo Scientific, Waltham, MA, USA) was used, and as detection antibody, biotin labeled αIFN-γclone B133.5 (M701b, Thermo Scientific, Waltham, MA, USA). As IFN-γ standard, recombinant human αIFN-γ (RIFNG100, Thermo Scientific, Waltham, MA, USA) was used. The assay was performed according standard ELISA procedures. To enable multiple cytokine detection in culture supernatants, the human Th1/Th2 11 plex Flowcytomix Multiplex (BMS810FFRTU, Bender MedSystems, eBioscience, San Diego, CA, USA) was used according to the protocol of the manufacture.

### 4.5. Flow Cytometric Analysis

JAK3, STAT5, phospho-STAT5, phospho-I3K, phospho-ERK1/2, mitogen-activated protein kinase (MAPK), cyclin D, and cyclin E, cells were first stained for cell surface markers after which the cells were fixed for 10 min at 37 °C using 2% paraformaldehyde, and permeabilized by incubating the cells in 90% methanol for 30 min on ice. Subsequently, the cells were stained with anti-JAK3, anti-STAT5, anti-phospho-STAT5, anti-phospho-Akt, anti-phospho-ERK1/2, anti-cyclin D, or anti-cyclin E. As secondary antibody, goat-anti-rabbit IgG Alexa 647 (A21245, Invitrogen, Thermo Fisher Scientific, Waltham, MA, USA) or goat-anti-mouse IgG Alexa 488 (A11029, Invitrogen, Thermo Fisher Scientific, Waltham, MA USA) was used. For the detection of phospho-STAT5, cells were stimulated with medium or 100 U/mL IL-2 for one hour at 37 °C prior to fixation. Cells were measured on a CyAn^TM^ ADP Analyzer equipped with a 488, 561, and 630 nm laser (Beckman Coulter, Brea, CA, USA), and analyzed with Summit 4.3.

### 4.6. Lipid Mediator Analysis of Abdominal Fluid

Abdominal fluid from five high-grade serous ovarian cancer patients and five control individuals was centrifuged to remove cells and debris. The supernatant was collected and stored at –80 °C until further analysis. Samples were prepared by thawing abdominal fluids and mixing 900 µL with 10 µL of internal standard solution and 900 µL of extraction buffer (citric acid: Na_2_HPO_4_, pH = 5.6). Solid phase extractions were done using an Extrahera liquid handling system (Biotage, Uppsala, Sweden) and Evolute Express ABN SPE cartridges (cartridge capacity: 60 mg, 3 mL sample volume, Biotage, Uppsala, Sweden). After conditioning (2.5 mL of methanol) and equilibration (2.5 mL of H_2_O) samples were loaded on the cartridges and washed with 2 mL of H_2_O/methanol (90:10). Lipid mediators were eluted in 2.5 mL methanol. As trap solution 30% glycerol in methanol (30 µL) was added to each eluate and methanol was evaporated under a nitrogen gas stream using a TurboVap LV evaporation system (Biotage, Uppsala, Sweden). Extracts were reconstituted in 70 µL methanol/water (6:1, *v*/*v*) and filtered by centrifugation for 2 min at 12,000× *g* using 0.1 µm polyvinylidenfluorid membrane spin filters (Merck Millipore Cooperation, Billerica, MA, USA). Quantitative analysis of lipid mediators was done using a liquid chromatography-mass spectrometry (LC-MS/MS) method previously described [43].

### 4.7. Statistics

GraphPad Prism software version 5 was used for statistical analysis. Error bars represent mean ± SD or SED, as indicated in the figure legends. Comparison of three or more groups was done on Log(Y) transformed data. One-way ANOVA with Bonferroni (significance level *p* = 0.05) post test were used to compare three or more groups with equal variances (determined by Bartlett’s test for equal variances). In the case of unequal variances, data were log-transformed. If variances were still different, non-parametric Kruskal-Wallis test with Dunns (significance level *p* = 0.05) post test was used. For the comparison of two groups, unpaired, two-tailed Student’s *t*-test was used. Differences were considered to be significant when * *p* < 0.05, ** *p* < 0.01 or *** *p* < 0.001.

## Figures and Tables

**Table 1:** Concentration of lipid intermediates in abdominal fluid of controls and ascites of ovarian cancer patients.

Sample (ng/mL)	Mean	SD	Mean	SD	Ratio
	Controls (*n* = 5)		Patients (*n* = 5)		Patients/Controls
5-HETE	85.26	81.8	289.69	58.2	3.4
EPA	101	16.4	169.51	81	1.7
4-HDoHE	38.66	40	120.22	20.8	3.1
13-HODE	24.34	6.1	109.87	64.4	4.5
9-HODE	6.07	3.6	74.4	46.3	12.3
9-HETE	2.75	2.4	65.47	35.3	23.8
13-KODE	1.34	0.8	45.9	29	34.3
5-HEPE	10.34	12.4	45.61	15	4.4
11-HETE	2.3	1.5	38.75	18.1	16.9
12-HETE	6.4	5.8	35.95	16.1	5.6
8-HETE	1.99	1.5	32.24	17.9	16.2
9-KODE	0.76	0.5	30.94	26.6	40.5
6-*trans*-LTB4	1.56	1.8	29.5	13.2	18.9
8-HDoHE	1.41	1.2	25.36	12	17.9
11-HDoHE	1.46	1.1	25.08	12.9	17.2
5,15-DiHETE	2.47	2.6	23.63	10	9.6
17-HDoHE	10.3	5	22.67	8.1	2.2
PGD2	0.85	0.7	20.57	8.1	24.3
PGD1	0.45	0.4	17.27	9.5	38.5
5-HETrE	3.05	3.4	16.5	7.2	5.4
8,15-DiHETE	0.26	0.2	16.31	8.5	63.8
15-HETE	10.05	4.8	15.14	4.5	1.5
15-KETE	0.92	0.6	12.28	7.3	13.3
5,6-DiHETE	0.58	0.7	10.54	4.7	18.2
14-HDoHE	2.14	0.8	7.83	2.5	3.7
9-HEPE	0.31	0.3	7.19	3.8	23.1
15-HETrE	1.2	0.4	6.65	2.3	5.6
5-KETE	0.57	0.8	5.8	2.7	10.2
12-HEPE	0.53	0.2	5.09	2.7	9.5
12-KETE	0.16	0.2	4.98	3	30.8
13-HOTrE	3.04	1.1	4.86	4.2	1.6
11-HEPE	0.25	0.2	4.79	2.6	19.4
10,17-DiHDoHE	0.06	0.1	4.15	2	65.1
5-iPF2a-VI	0.25	0.2	3.1	1.4	12.6
8-HEPE	0.23	0.2	3.09	1.8	13.7
8-HETrE	0.26	0.2	2.72	1	10.5
PGE1	0.19	0.1	2.46	1.1	13.1
9,10,13-TriHOME	0.38	0.2	2.07	1.1	5.5
9,12,13-TriHOME	0.48	0.3	1.87	0.9	3.9
18-HEPE	0.15	0.1	1.86	0.8	12.1
LXB4	0.08	0.1	1.83	0.9	22.5
15-HEPE	0.68	0.4	1.82	0.7	2.7
9-HOTrE	0.36	0.2	1.62	1.3	4.5
12,13-DiHOME	4.57	1	1.53	0.9	0.3
13-HOTrE(gamma)	0.41	0.1	1.37	0.4	3.3
EKODE	0.85	0.2	1.36	0.8	1.6
11-HEDE	0.29	0.1	1.36	0.8	4.7
9,10-DiHOME	7.01	2.2	1.35	0.7	0.2
20-COOH-LTB4	0	0	1.16	0.8	-
6-keto-PGF1a	12.78	10.9	1.13	0.6	0.1
8(9)-EpETrE	0.05	0.1	1.1	0.4	20
17(18)-EpETE	0.18	0.2	1.05	0.3	5.7
15-HEDE	0.1	0	0.82	0.4	8.1
12-HHTrE	3.29	4.7	0.78	0.5	0.2
8-isoPGE2	0.21	0.1	0.76	0.1	3.7
LTB4	0.06	0	0.67	0.4	11.8
17,18-DiHETE	1.01	0.2	0.67	0.2	0.7
14,15-DiHETrE	0.39	0	0.65	0.1	1.7
PGE2	1.07	1.2	0.64	0.3	0.6
19-HETE	0.6	0.2	0.64	0	1.1
20-HETE	0.28	0.1	0.63	0.1	2.3
11,12-DiHETrE	0.31	0.1	0.51	0	1.6
14,15-DiHETE	0.23	0	0.5	0.2	2.1
7-Maresin-1	0	0	0.49	0.3	-
12(13)-EpOME	0.47	0.1	0.48	0.2	1
19,20-DiHDPA	0.3	0	0.39	0.1	1.3
5,6-DiHETrE	0.18	0	0.31	0.1	1.8
7,17-hydroxy-DPA	0.03	0	0.28	0.1	9.3
11-keto-TXB2	0.03	0	0.27	0.1	10.2
9(10)-EpOME	0.22	0.1	0.26	0.1	1.2
LTE4	0.12	0.1	0.2	0.1	1.7
iPF2a-IV	0	0	0.2	0.1	48.3
PGB2	0.34	0.4	0.17	0.1	0.5
8,9-DiHETrE	0.11	0	0.17	0	1.5
11B-PGF2a	0.29	0.4	0.11	0	0.4
8-iso-PGF2a	0.04	0	0.07	0	2
15-KEDE	0.01	0	0.07	0	5.5
12(13)EpODE	0.02	0	0.05	0	2.5
15-deoxy-delta-12,14-PGJ2	0.01	0	0	0	0
LTB3	0	0	0	0	-
PGD3	0	0	0	0	-
delta-12-PGJ2	0	0	0	0	-
17-RvD1	0	0	0	0	-
11(12)-EpETrE	0	0	0	0	-
PGF2a	6.22	8.1	0	0	-
PGE3	0	0	0	0	-
PGJ2	0	0	0	0	-
RvD1	0	0	0	0	-
RvD2	0	0	0	0	-
14(15)-EpETE	0	0	0	0	-
14(15)-EpETrE	0	0	0	0	-
16(17)-EpDPE	0	0	0	0	-
19(20)-EpDPE	0	0	0	0	-
5(6)-EpETrE	0	0	0	0	-
9-KOTrE	0	0	0	0	-
20-OH-LTB4	0	0	0	0	-
LXA4	0	0	0	0	-
LXA5	0	0	0	0	-
tetranor-PGDM	0	0	0	0	-
13,14-dihydro-15-keto-PGE2	0.01	0	0	0	0
8-iso-PGF3a	0	0	0	0	-
11-keto-TXB3	0	0	0	0	-

## Abbreviations

T_Regs_	Regulatory T cells
TIL	Tumor infiltrating lymphocytes
PBLs	Peripheral blood lymphocytes
PBMCs	Peripheral blood mononuclear cells
MNCs	Mononuclear cells
LOX	Lipooxygenase
Cpm	Counts per minute
IFN	Interferon
TGF	Tumor growth factor
TNF	Tumor necrosis factor
IL	Interleukin
IL-2R	Interleukin 2 receptor
JAK3	Janus kinase 3
STAT5	Signal transducer and activator of transcription 5
MAPK	Mitogen-activated kinase
FACS	Fluorescent activated cell sorting
PPAR	Peroxisome proliferator-activated receptor
NFAT	Nuclear factor of activated T cells
